# Lower Dietary and Circulating Vitamin C in Middle- and Older-Aged Men and Women Are Associated with Lower Estimated Skeletal Muscle Mass

**DOI:** 10.1093/jn/nxaa221

**Published:** 2020-08-27

**Authors:** Lucy N Lewis, Richard P G Hayhoe, Angela A Mulligan, Robert N Luben, Kay-Tee Khaw, Ailsa A Welch

**Affiliations:** Department of Epidemiology and Public Health, Norwich Medical School, Faculty of Medicine and Health Sciences, University of East Anglia, Norwich, United Kingdom; Department of Epidemiology and Public Health, Norwich Medical School, Faculty of Medicine and Health Sciences, University of East Anglia, Norwich, United Kingdom; MRC Epidemiology Unit, University of Cambridge School of Clinical Medicine, Cambridge, United Kingdom; Department of Public Health and Primary Care, Institute of Public Health, University of Cambridge, Cambridge, United Kingdom; Department of Public Health and Primary Care, Institute of Public Health, University of Cambridge, Cambridge, United Kingdom; Department of Epidemiology and Public Health, Norwich Medical School, Faculty of Medicine and Health Sciences, University of East Anglia, Norwich, United Kingdom

**Keywords:** sarcopenia, skeletal muscle, frailty, vitamin C, ascorbic acid

## Abstract

**Background:**

Age-related loss of skeletal muscle mass contributes to poor outcomes including sarcopenia, physical disability, frailty, type 2 diabetes, and mortality. Vitamin C has physiological relevance to skeletal muscle and may protect it during aging, but few studies have investigated its importance in older populations.

**Objectives:**

We aimed to investigate cross-sectional associations of dietary and plasma vitamin C with proxy measures of skeletal muscle mass in a large cohort of middle- and older-aged individuals.

**Methods:**

We analyzed data from >13,000 men and women in the European Prospective Investigation into Cancer and Nutrition–Norfolk cohort, aged 42–82 y. Fat-free mass (FFM), as a proxy for skeletal muscle mass, was estimated using bioelectrical impedance analysis and expressed as a percentage of total mass (FFM%) or standardized by BMI (FFM_BMI_). Dietary vitamin C intakes were calculated from 7-d food diary data, and plasma vitamin C was measured in peripheral blood. Multivariable regression models, including relevant lifestyle, dietary, and biological covariates, were used to determine associations between FFM measures and quintiles of dietary vitamin C or insufficient compared with sufficient plasma vitamin C (<50 μmol/L and ≥50 μmol/L).

**Results:**

Positive trends were found across quintiles of dietary vitamin C and FFM measures for both sexes, with interquintile differences in FFM% and FFM_BMI_ of 1.0% and 2.3% for men and 1.9% and 2.9% for women, respectively (all *P* < 0.001). Similarly, FFM% and FFM_BMI_ measures were higher in participants with sufficient than with insufficient plasma vitamin C: by 1.6% and 2.0% in men, and 3.4% and 3.9% in women, respectively (all *P* < 0.001). Associations were also evident in analyses stratified into <65-y and ≥65-y age groups.

**Conclusions:**

Our findings of positive associations, of both dietary and circulating vitamin C with measures of skeletal muscle mass in middle- and older-aged men and women, suggest that dietary vitamin C intake may be useful for reducing age-related muscle loss.

## Introduction

Sarcopenia is characterized by a progressive and generalized loss of skeletal muscle mass and strength (1–4). Increasing age is a well-recognized risk factor for sarcopenia such that worldwide the condition affects >50 million people over the age of 50 y (2, 3). Whereas maintenance of strength and function is recognized as important for preventing functional limitations, physical disability, and loss of mobility, less recognized are the metabolic disturbances associated with loss of skeletal muscle mass (4–8). These metabolic disturbances include altered utilization of amino acids, glucose, and fatty acids, as well as contributions to the onset of obesity and type 2 diabetes (4–8). Sarcopenia and age-related skeletal muscle loss are also key contributors to frailty. Despite growing appreciation that reducing loss of skeletal mass and function with age is important, current options for prevention are limited.

The etiology of sarcopenia is multifactorial, with several contributory mechanisms including endocrine causes, age-related changes in circulating cytokines, production of reactive oxygen species (ROS), immobility, and low intakes of protein (1). ROS, which are produced during oxidative metabolism in muscle, and from age-related mitochondrial dysfunction, induce cellular damage in muscle, as does the age-related increase in circulating concentrations of inflammatory cytokines (9–11). Vitamin C, a water-soluble vitamin obtained by consumption of fruits, vegetables, and their products in the diet, has several mechanistic functions relevant to skeletal muscle metabolism and physiology, which could prevent age-related loss of skeletal muscle. The mechanisms for vitamin C in skeletal muscle physiology include synthesis of carnitine and collagen and recent animal studies have further elaborated the role of vitamin C deficiency (12–15). Because vitamin C is an electron donor, this may reduce oxidative damage to muscle as well as reducing the concentrations of inflammatory cytokines in the circulation (10, 11). Vitamin C deficiency, also known as scurvy, is identified by circulating concentrations of ascorbic acid <11.4 μmol/L, with concentrations <50 μmol/L considered insufficient. Evidence from validation studies indicates that these circulating concentrations are appropriate biomarkers of dietary vitamin C in epidemiological studies (16–19).

Despite knowledge of the mechanisms by which vitamin C can affect skeletal muscle physiology during aging, the importance of vitamin C in relation to skeletal muscle mass has not been extensively studied. We are unaware of previous epidemiological studies where both dietary and plasma vitamin C have been studied in relation to indexes of skeletal muscle mass in both sexes and both middle and older age, although some individual studies have been performed previously (13, 20–25). Given the relevance of vitamin C to skeletal muscle physiology and the lack of previous research on the importance of vitamin C to the sarcopenic risk factor of skeletal muscle mass, the purpose of this study was to investigate the associations of dietary and plasma vitamin C with fat-free mass (FFM) (as a proxy measure of skeletal muscle mass) in a large general population cohort of middle- and older-aged men and women. We thus investigated the cross-sectional associations between indexes of FFM and dietary intake, as well as circulating concentrations, of vitamin C, in a population of 13,000 free-living men and women in the United Kingdom in middle and older age. We also sought to determine the associations in those older and younger than 65 y of age.

## Methods

### Study population

**FIGURE 1:**
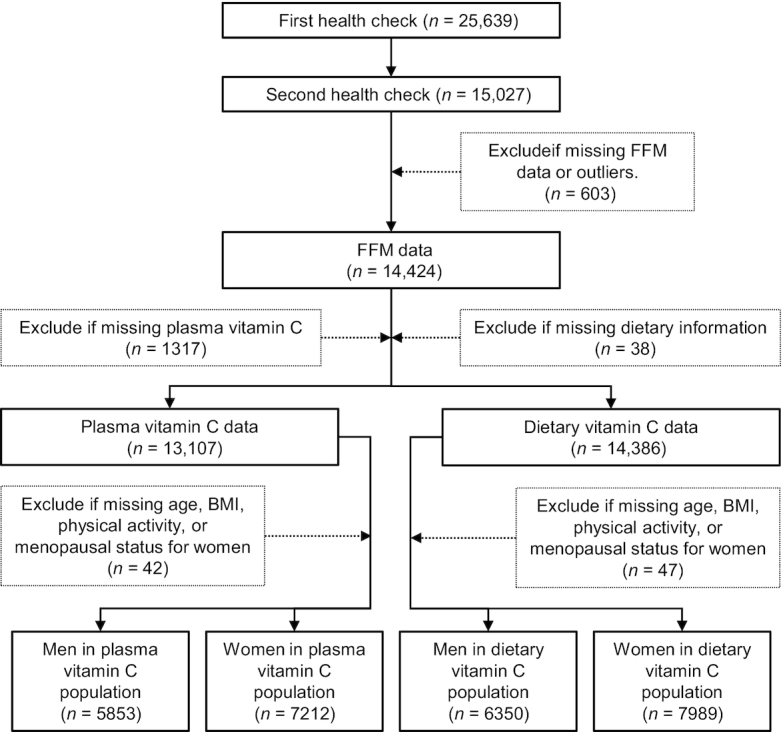
Flowchart of participants through the study. FFM, fat-free mass.

The European Prospective Investigation into Cancer and Nutrition (EPIC) study is a prospective cohort involving >500,000 study participants from 10 European countries, initially designed to investigate the relations between diet and cancer. Additional outcomes have been examined within the UK EPIC-Norfolk subcohort, consisting of 25,639 men and women aged 40–79 y who attended baseline health checks between 1993 and 1997 (26). A second, follow-up, health check was attended by 17,304 participants aged 42–82 y between 1997 and 2000 in which measures of body composition were made. Our analyses were limited to 6350 men and 7989 women with complete data for dietary vitamin C analyses and 5853 men and 7212 women with complete data for plasma vitamin C analyses (see **Figure 1**).

### Dietary assessment

Dietary intake of each participant was assessed using a 7-d food diary. The participants recorded all food and drink consumed within a 7-d period, including details of portion sizes. This method has been found to be more accurate than FFQs in estimating dietary nutrient intake when compared with weighted records (17, 27). The Data Into Nutrients for Epidemiologic Research (DINER) software used to document the dietary information provided by the 7-d food diaries and convert it into nutrient quantities has been described previously (28). Nondietary data were collected through health and lifestyle questionnaires at each health check that included questions on smoking, alcohol consumption, social class, occupational history, past history of disease, short family history of main disease endpoints, a short section on exercise, and reproductive history for women (26).

### Body composition

Each participant had their height and weight measured using standardized methodology (26) at both health checks: height was measured to the nearest millimeter without shoes, using a freestanding stadiometer; weight was measured to the nearest 0.2 kg without shoes and in light clothing, using digital scales. Body fat was measured at the second health check using a bioelectrical impedance analysis (BIA) machine (TANITA Body Fat Monitor/Scale TBF-531), with individuals in the standing position, from which FFM (kg) was calculated as a proxy measure of skeletal muscle mass. The percentage fat-free mass (FFM%; FFM/total mass × 100) and fat-free mass standardized by BMI (FFM_BMI_; FFM/BMI) were calculated, as these are previously established and recommended indexes which scale for the effects of body size on the proportion of FFM (2).

### Plasma vitamin C

To obtain vitamin C plasma concentrations, nonfasting blood samples were taken from participants using venipuncture. Blood samples were protected from light and without delay the plasma fraction was isolated and immediately stabilized with metaphosphoric acid. Stabilized samples were stored at −70°C and within 1 wk the plasma vitamin C concentration was estimated by fluorometric assay (29, 30).

### Statistical methods

STATA statistical software version 15 (Stata Corp.) was used to examine the relation between vitamin C and FFM. Sex differences for variables used in our analysis models were tested by independent-sample *t* test for continuous or chi-square test for categorical variables. Our analyses combined dietary data from health check 1, with covariate, body composition, and plasma vitamin C data from health check 2. Univariate regression was first used to investigate the differences in FFM across sex-specific quintiles (Qs) of dietary vitamin C intake. Owing to the established sex differences in body composition, all the analyses were stratified by sex. A multivariable model was then tested, incorporating biological [age; menopausal status; hormone replacement therapy (HRT) status; corticosteroid use; and statin use], lifestyle (smoking status classified as current, former, or never; social class classified by 6 categories; and physical activity status classified as inactive, moderately inactive, moderately active, and active), and dietary covariates (total energy; protein intake as a percentage of total energy; number of days participant filled in the food diary; and the ratio of energy intake to estimated energy requirement). In order to test for trends we used the median values for quintiles as a continuous variable. We also calculated the adjusted values for FFM and used these to determine the percentage differences in FFM between specific vitamin C quintiles. ANCOVA was used to determine whether these differences were statistically significant with a *P* value < 0.05. The European Food Safety Authority (EFSA) consider a serum ascorbic acid concentration <11.4 μmol/L as deficient, <50 μmol/L as insufficient, and ≥50 μmol/L as sufficient (16, 31). Because the number classified as deficient in this cohort was small, we performed the plasma vitamin C analyses using the broader categorization of insufficiency (plasma vitamin C <50 μmol/L; *n* = 2035 in men and *n* = 1212 in women) compared with sufficiency (plasma vitamin C ≥50 μmol/L; *n* = 3818 in men and *n*  = 6000 in women). Similarly to the diet analyses, we first tested an unadjusted regression model to identify any differences in indexes of FFM according to categories of plasma vitamin C concentration. We then tested a model adjusted for age, menopausal status, HRT status, statin use, corticosteroid use, smoking, physical activity status, and social class. All models were repeated stratifying by age group (<65 y or ≥65 y). We performed additional analyses to compare the main models relating dietary vitamin C to FFM indexes, calculated with vitamin C contributions from food and drinks only, with models which also included vitamin C contributions from supplements, calculated using data from the vitamin and mineral supplement database (ViMiS) developed for EPIC-Norfolk (32). Univariate regression was used to test the association between dietary and plasma vitamin C. Exclusions were made where participants had missing values for any variables included in the multivariable model (see Figure 1). Those who had extreme BIA values (>1000 or <300 ohms), and those who had an FFM <25 kg or a BMI ≥36 kg/m^2^, were excluded because estimating FFM from BIA including these values would be inaccurate (33). Women who were missing menopausal status had their status recoded to postmenopausal if >55 y and ever users of HRT, or premenopausal if <50 y and never users of HRT. Any participants missing data for smoking status were recoded as former smokers because there was higher prevalence of smoking in this cohort than in the UK population as a whole. All models were defined a priori using evidence from previous research, and thus *P* values < 0.05 were considered statistically significant in individual analyses.

## Results

**Table 1** and **Supplemental Table 1** summarize participant characteristics for men and women; data are expressed as means ± SDs for continuous variables or percentages for categorical variables. There were 6350 men and 7989 women with complete data for dietary vitamin C analyses; for plasma vitamin C analyses, there were 5853 men and 7212 women. Intakes of vitamin C ranged from 36.6 ± 9.33 mg/d for men in Q1 to 170 ± 44.8 mg/d in Q5 and from 38.9 ± 9.41 mg/d for women in Q1 to 171 ± 43.4 mg/d in Q5, and mean interquintile differences were 133 and 132 mg/d, respectively. In this study 0.9% men and 0.2% of women were classified as deficient according to plasma concentrations <11.4 μmol/L and 34.7% of men and 16.8% of women were classified as insufficient (plasma concentrations <50 μmol/L).

**TABLE 1:** Participant characteristics of the European Prospective Investigation into Cancer and Nutrition–Norfolk cohort population stratified by sex for the dietary vitamin C and the plasma vitamin C group^1^

	Dietary vitamin C (*n* = 14,339)		Plasma vitamin C (*n* = 13,065)	
Characteristics	Men (*n* = 6350)	Women (*n* = 7989)	Men (*n* = 5853)	Women (*n* = 7212)
Age, y	62.9 ± 9.03	61.5 ± 9.04	62.9 ± 8.98	61.5 ± 9.01
BMI, kg/m^2^	26.7 ± 3.05	26.1 ± 3.73	26.7 ± 3.02	26.1 ± 3.70
Weight, kg	80.9 ± 10.8	67.7 ± 10.4	80.9 ± 10.7	67.6 ± 10.4
Height, cm	174 ± 6.61	161 ± 6.12	174 ± 6.60	161 ± 6.13
FFM%	76.7 ± 5.78	60.9 ± 8.25	76.7 ± 5.74	61.0 ± 8.20
FFM_BMI_, kg/m^2^	2.33 ± 0.257	1.58 ± 0.259	2.33 ± 0.256	1.59 ± 0.258
Vitamin C intake, mg/d	89.8 ± 50.7	93.7 ± 50.1	89.9 ± 50.5	94.0 ± 50.3
Plasma vitamin C, μmol/L	—	—	56.9 ± 21.3	68.9 ± 24.5
Protein, g/d	83.4 ± 17.6	66.2 ± 13.7	83.5 ± 17.6	66.3 ± 13.7
Protein, % energy	14.8 ± 2.40	15.5 ± 2.77	14.8 ± 2.39	15.5 ± 2.78
Energy intake, kcal/d	2286 ± 500	1735 ± 378	2289 ± 501	1736 ± 379
Smoking				
Current	8.54 (542)	8.71 (696)	8.46 (495)	8.72 (629)
Former	55.5 (3524)	31.9 (2551)	55.6 (3254)	32.1 (2312)
Never	34.0 (2284)	59.4 (4742)	35.9 (2104)	59.2 (4271)
Physical activity				
Inactive	27.3 (1736)	25.9 (2070)	26.8 (1566)	25.4 (1829)
Moderately inactive	25.1 (1595)	32.5 (2600)	24.9 (1458)	32.9 (2374)
Moderately active	25.0 (1590)	24.2 (1933)	25.4 (1485)	24.1 (1737)
Active	22.5 (1429)	17.3 (1386)	23.0 (1344)	17.6 (1272)
Corticosteroid use				
Current	4.16 (264)	5.09 (407)	4.05 (237)	5.06 (365)
Menopausal status				
Premenopausal	—	5.95 (475)	—	5.92 (427)
Perimenopausal <1 y	—	3.33 (266)	—	3.30 (238)
Perimenopausal 1–5 y	—	17.5 (1399)	—	17.4 (1256)
Postmenopausal >5 y	—	73.2 (5849)	—	73.4 (5291)
HRT use				
Current	—	21.3 (1704)	—	21.4 (1543)
Former	—	17.9 (1431)	—	17.9 (1288)
Never	—	60.8 (4854)	—	60.8 (4381)
Statins use				
Yes	5.46 (347)	3.63 (290)	5.43 (318)	3.56 (257)
Days participant filled in diary, *n*	6.75 ± 1.16	6.81 ± 1.01	6.75 ± 1.15	6.81 ± 1.01
Vitamin C supplementation				
Yes	34.5 (695)	39.8 (1425)	34.1 (633)	39.8 (1281)
Social class				
Professional	8.24 (523)	6.83 (546)	8.18 (479)	6.74 (486)
Managerial	40.7 (2587)	36.9 (2950)	40.9 (2395)	37.0 (2668)
Skilled nonmanual	12.6 (797)	19.4 (1554)	12.4 (723)	19.6 (1413)
Skilled manual	22.4 (1422)	19.7 (1577)	22.4 (1312)	19.8 (1426)
Semiskilled	12.5 (781)	11.9 (950)	12.4 (727)	11.9 (855)
Nonskilled	2.35 (149)	3.34 (267)	2.27 (133)	3.24 (234)

1 Values are means ± SDs or % (*n*). Differences between men and women for all characteristics had *P* values < 0.01, according to *t* test for continuous or chi-square test for categorical variables. FFM_BMI_, fat-free mass standardized by BMI; FFM%, fat-free mass percentage; HRT, hormone replacement therapy.

**FIGURE 2:**
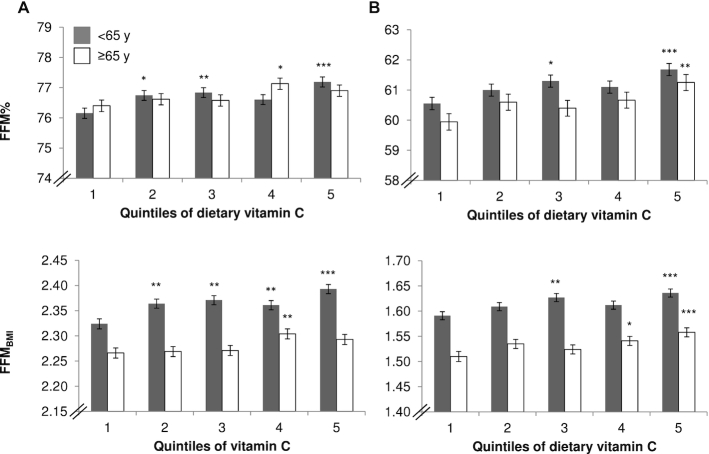
Adjusted FFM measures for individuals of the European Prospective Investigation into Cancer and Nutrition–Norfolk cohort stratified by sex [men (A); women (B)], age group, and quintiles of dietary vitamin intake (*n* = 14,339). ^*,**,***^Significant difference with quintile 1, according to ANCOVA: *P < 0.05; **P < 0.01; ***P < 0.001. Adjusted model includes age, total energy, protein intake as a percentage of total energy, estimated energy requirement, smoking status, physical activity, corticosteroid use, menopausal status, hormone replacement therapy use, statins use, number of days participant filled in the food diary, and social class. Values are presented as means with SEMs as error bars. FFM, fat-free mass; FFM_BMI_, fat-free mass standardized by BMI; FFM%, fat-free mass percentage.

Positive associations were found between dietary vitamin C and FFM% in both men and women (*P* < 0.001, *n* = 6350 men; and *P* < 0.001, *n* = 7989 women) after adjustment for covariates, with significant interquintile differences (Q5 compared with Q1) in FFM% of +1.0% (*P* < 0.001, *n* = 6350) in men and +1.9% (*P* < 0.001, *n* = 7989) in women (see **Table 2**). Similar associations were also found between vitamin C and FFM_BMI_ (*P* < 0.001 for both men and women) after adjustment for covariates; and interquintile differences were significant (see **Figure 2**), with Q5 compared with Q1 differences in FFM_BMI_ of +2.3% in men and +2.9% in women (all *P* < 0.01). Results of additional analyses including the contribution of vitamin C from supplements showed positive associations between total vitamin C intakes and FFM measures and no substantial differences to the results of the nonsupplement models presented here in full.

**TABLE 2:** Associations between Qs of dietary vitamin C and skeletal muscle mass in men and women aged 42–82 y^1^

	Vitamin C		FFM%		FFM_BMI_	
	Mean ± SD	Median	Unadjusted	Adjusted	Unadjusted	Adjusted
Men (*n* = 6350)						
Q1 (*n* = 1270)	36.6 ± 9.33	38.5	76.2 ± 0.169	76.3 ± 0.128	2.27 ± 0.007	2.30 ± 0.007
Q2 (*n* = 1270)	57.9 ± 5.38	57.7	76.9 ± 0.161**	76.7 ± 0.124*	2.32 ± 0.007***	2.32 ± 0.007*
Q3 (*n* = 1270)	78.4 ± 6.64	78.3	76.8 ± 0.159*	76.7 ± 0.124*	2.33 ± 0.007***	2.33 ± 0.007**
Q4 (*n* = 1270)	107 ± 10.1	106	76.8 ± 0.166**	76.8 ± 0.125**	2.34 ± 0.007***	2.34 ± 0.007***
Q5 (*n* = 1270)	170 ± 44.8	157	76.9 ± 0.156***	77.1 ± 0.125***	2.36 ± 0.007***	2.35 ± 0.007***
Q5–Q1 difference^2^	—	—	0.694 (0.244, 1.14)	0.763 (0.404, 1.121)	0.088 (0.068, 0.108)	0.052 (0.032, 0.071)
% difference^3^	—	—	0.910	1.00	3.88	2.25
*P*-trend	—	—	0.024	<0.001	<0.001	<0.001
Women (*n* = 7989)						
Q1 (*n* = 1598)	38.9 ± 9.41	40.6	60.1 ± 0.217	60.3 ± 0.166	1.55 ± 0.007	1.56 ± 0.006
Q2 (*n* = 1598)	62.6 ± 5.93	62.5	61.0 ± 0.208**	60.9 ± 0.162*	1.58 ± 0.007***	1.58 ± 0.006*
Q3 (*n* = 1598)	83.9 ± 6.75	83.6	61.0 ± 0.207**	60.9 ± 0.162*	1.59 ± 0.006***	1.59 ± 0.006**
Q4 (*n* = 1598)	112 ± 9.88	111	61.0 ± 0.198**	60.9 ± 0.163*	1.59 ± 0.006***	1.58 ± 0.006**
Q5 (*n* = 1597)	171 ± 43.4	159	61.5 ± 0.201***	61.5 ± 0.164***	1.61 ± 0.006***	1.61 ± 0.006***
Q5–Q1 difference^2^	—	—	1.40 (0.83, 1.97)	1.14 (0.680, 1.61)	0.064 (0.046, 0.082)	0.050 (0.028, 0.062)
% difference^3^	—	—	2.33	1.90	4.17	2.89
*P*-trend	—	—	<0.001	<0.001	<0.001	<0.001

1 Values are means ± SEMs. The *P*-trend was calculated using ANCOVA. Adjusted model includes age, total energy, protein intake as a percentage of total energy, estimated energy requirement, smoking status, physical activity, corticosteroid use, menopausal status, hormone replacement therapy use, statins use, number of days participant filled out the food diary, and social class. ^*,**,***^Significant difference with quintile 1, according to ANCOVA: *P < 0.05; **P < 0.01; ***P < 0.001. FFM_BMI_, fat-free mass standardized by BMI; FFM%, fat-free mass percentage; Q, quintile.

2 Q5–Q1 calculates the absolute difference between the means of Q5 and Q1, with 95% CIs.

3 % difference calculates the percentage difference between the means of Q5 and Q1.

**FIGURE 3:**
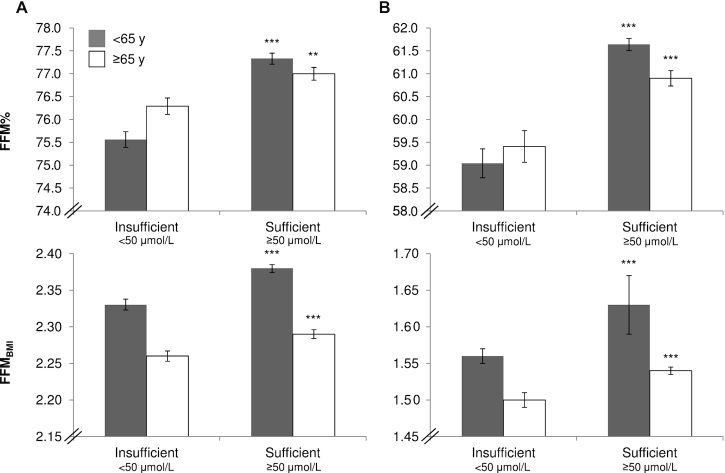
Adjusted FFM measures for individuals of the European Prospective Investigation into Cancer and Nutrition–Norfolk cohort stratified by sex [men (A); women (B)], age group, and plasma categories of vitamin C (<50 μmol/L and ≥50 μmol/L) (*n* = 13,065). ^**,***^Significant difference with insufficient, according to ANCOVA: **P < 0.01; ***P < 0.001. Adjusted model includes age, smoking status, physical activity, corticosteroid use, menopausal status, hormone replacement therapy use, statins use, and social class. Values are presented as means with SEMs as error bars. Men <65 y: insufficient, *n* = 1038; sufficient, *n* = 2171. Men ≥65 y: insufficient, *n* = 997; sufficient, *n* = 1647. Women <65 y: insufficient, *n* = 668; sufficient, *n* = 3745. Women ≥65 y: insufficient, *n* = 544; sufficient, *n* = 2255. FFM, fat-free mass; FFM_BMI_, fat-free mass standardized by BMI; FFM%, fat-free mass percentage.

Significant differences in FFM% were found between individuals with sufficient as opposed to insufficient plasma vitamin C concentrations (*P* < 0.001, *n* = 5853 men; and *P* < 0.001, *n* = 7212 women) after adjustment for covariates (see **Table 3**). In the adjusted model, men who had sufficient plasma vitamin C concentrations (*n* = 3818) had a mean FFM% 1.6% higher than men with insufficient concentrations (*n* = 2035); in women the difference was 3.4% (*n* = 6000 sufficient compared with *n* = 1212 insufficient). Similarly, significant differences in FFM_BMI_ were found between individuals with sufficient as opposed to insufficient plasma vitamin C concentrations (*P* < 0.001 for both men and women) after adjustment for covariates. The difference between sufficient and insufficient individuals was +2.0% for FFM_BMI_ in men and +3.9% in women. In age-stratified analyses (<65 y and ≥65 y) (Figures 2, **3**, Supplemental Tables 1–**3**), although the baselines for FFM measures differed between the age groups, similar significant trends to those found in all-age analyses were evident for dietary or plasma vitamin C and measurements of FFM.

**TABLE 3:** Associations between plasma vitamin C concentration (insufficient: <50 μmol/L and sufficient: ≥50 μmol/L) and skeletal muscle mass in men and women aged 42–82 y^1^

	Dietary vitamin C		Plasma vitamin C		FFM%		FFM_BMI_	
Vitamin C categories	Mean ± SD	Median	Mean ± SD	Median	Unadjusted	Adjusted	Unadjusted	Adjusted
Men (*n* = 5853)								
Insufficient (*n* = 2035)	72.9 ± 39.7	63.4	35.9 ± 10.8	39.0	75.9 ± 0.131	75.9 ± 0.126	2.29 ± 0.006	2.30 ± 0.006
Sufficient (*n* = 3818)	99.0 ± 53.3	88.0	68.1 ± 16.5	64.0	77.2 ± 0.090	77.2 ± 0.092	2.35 ± 0.004	2.34 ± 0.004
Absolute difference^2^	—	—	—	—	1.29 (0.980, 1.59)	1.25 (0.943, 1.56)	0.060 (0.046, 0.073)	0.047 (0.033, 0.061)
% difference^3^	—	—	—	—	1.70	1.65	2.61	2.05
*P* value	—	—	—	—	<0.001	<0.001	<0.001	<0.001
Women (*n* = 7212)								
Insufficient (*n* = 1212)	73.1 ± 40.6	63.8	37.1 ± 10.1	40.0	59.3 ± 0.260	59.3 ± 0.235	1.53 ± 0.008	1.54 ± 0.007
Sufficient (*n* = 6000)	98.2 ± 51.0	88.4	75.4 ± 21.4	72.0	61.3 ± 0.103	61.3 ± 0.104	1.60 ± 0.003	1.60 ± 0.003
Absolute difference^2^	—	—	—	––	2.03 (1.53, 2.54)	2.03 (1.53, 2.54)	0.068 (0.052, 0.084)	0.060 (0.044, 0.076)
% difference^3^	—	—	—	—	3.43	3.43	4.43	3.91
*P* value	—	—	—	—	<0.001	<0.001	<0.001	<0.001

1 Values are means ± SEMs. *P* values were calculated using ANCOVA comparing the 2 categories. Adjusted model includes age, smoking status, physical activity, corticosteroid use, menopausal status, hormone replacement therapy use, statins use, and social class. FFM_BMI_, fat-free mass standardized by BMI; FFM%, fat-free mass percentage.

2 Absolute difference calculates the absolute difference between means of the 2 categories, with 95% CIs.

3 % difference calculates the percentage difference between means of the 2 categories.

Results from the univariate regression between dietary and plasma vitamin C in the whole cohort population showed that for every 1-mg increase in vitamin C intake per day there was an increase in plasma vitamin C concentration of 0.478 μmol/L (*P* < 0.001, *n* = 13,033). The rate was greater in men than in women: 0.647 μmol · L^−1^ · mg^−1^ increase in men (*P* < 0.001, *n* = 5832) and 0.392 μmol · L^−1^ · mg^−1^ increase in women (*P* < 0.001, *n* = 7201).

**FIGURE 4:**
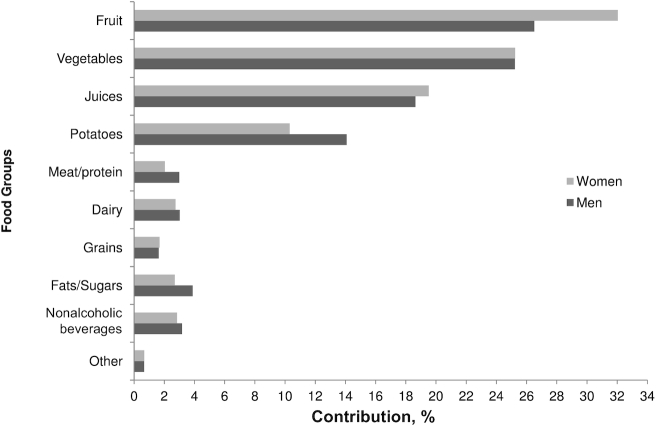
Percentage contribution of food groups to vitamin C intake of individuals of the European Prospective Investigation into Cancer and Nutrition–Norfolk cohort. Men: fruit (26.5%); vegetables (25.2%); juice (18.6%); potatoes (14.1%); meat/protein (3.0%); dairy (3.0%); grains (1.6%); fats/sugars (3.9%); nonalcoholic beverages (3.2%); other (0.7%). Women: fruit (32.0%); vegetables (25.2%); juices (19.5%); potatoes (10.3%); meat/protein (2.0%); dairy (2.7%); grains (1.7%); fats/sugars (2.7%); nonalcoholic beverages (2.8%); other (0.7%). Fruit includes apples, apricots, avocado, bananas, berries, blueberries, citrus, figs/dates, grapes, melon, mixed fruits, other, peach/nectarines, pears, and plums. Vegetables include herbs, brassica (cabbage), carrots, cauliflower, cucumber, green beans, other, peas, salad, and tomato. Juices include juices and citrus juice. Meat/protein includes eggs, game, meat products, poultry, red meat, red meat dishes, red meat products, and offal. Dairy includes cheeses, cream, milk (semiskimmed, skimmed, full), and yogurts. Grains include bread, oat cereal, cereals, pasta, and rice. Fats/sugars includes savory biscuits, sweet biscuits, cake, confectionery, fats, jam, syrups, pastry and batter products, cereal puddings, milk-based puddings, savory sauces, sweet sauces, savory foods, and sugars. Nonalcoholic beverages include coffee, tea, and squash. Other includes beans, bean dishes, dressings, diet meals, meals, nuts, seeds, pickles, and soups.

The greatest percentage contributions of different food groups to the daily vitamin C intake of the population were from fruits, vegetables, potatoes, and fruit juices (84.4% of the total intake in men and 87.1% in women) (**Figure 4**). Overall, the greatest contribution to vitamin C intake was from fruit intake (26.5% in men and 32% in women), followed by vegetables (25.2% for both sexes).

## Discussion

To our knowledge, this is the first study assessing the relation of dietary and circulating vitamin C with the sarcopenic risk factor of loss of skeletal muscle mass in a large UK cohort of both men and women of middle and older age. Our results show significant positive associations between dietary vitamin C intake and measures of FFM (as proxies for skeletal muscle mass) using multivariable regression models, adjusted for known lifestyle and biological covariates. The magnitude of differences seen with the indexes of FFM between individuals with intakes of vitamin C in the lowest and highest quintiles was greater for FFM_BMI_ than for FFM%, with the largest difference of 2.9% seen in FFM_BMI_ of women. Importantly, our findings with dietary vitamin C intake values derived from self-reported food diary data were reinforced and validated by our analyses using biomarker data. These showed similar trends and strong associations of circulating vitamin C and indexes of FFM, with statistically significant differences between sufficient and insufficient concentrations, with the largest difference of 3.9% seen in FFM_BMI_ of women. Although associations were significant for both sexes, the scale of the associations was greater in women than in men. Similar associations were seen in age-stratified analyses for those above and below 65 y of age, although the magnitude of differences in FFM measures varied by age group. Previous studies have shown that people over the age of 50 y experience a 0.5%–1% loss of muscle mass per year (34), thus suggesting, by comparison, that the magnitude of the differences seen in our analyses could be of clinical importance.

Only a small number of previous studies have investigated associations between either dietary or circulating vitamin C concentrations and indexes of skeletal muscle mass or function (20–25).

Of the 3 previous cohort studies investigating indexes of skeletal muscle mass and vitamin C, 2 found significant differences in skeletal muscle mass measures ranging from 1% to 3.2% between extreme quintiles of vitamin C intake in women, and differences ≤3.5% across quartiles of vitamin C intake in men and women over a follow-up period of 2.6 y (23, 25); the third found no associations between skeletal muscle mass measures and plasma vitamin C concentrations (20). Three other studies found no association between intake of vitamin C and prevalence of sarcopenia (35–37). All these previous studies investigating either dietary or circulating vitamin C and FFM, muscle function, or sarcopenia were in smaller populations than in our study and none investigated associations in a mixed population stratified by sex (20–25, 35–37).

### Mechanisms

The mechanistic roles for vitamin C in skeletal muscle physiology include the synthesis of carnitine and collagen. These are important because collagen is a key structural component of skeletal muscle cells and tendons, and carnitine is essential for metabolism of long-chain fatty acids during physical activity (14, 15). Animal studies have further elucidated the mechanisms relating to skeletal muscle atrophy, and the morphological changes caused by deficiency of dietary vitamin C. The main drivers appear to be upregulation of the ubiquitin ligases atrogin1/muscle atrophy F-box and muscle RING-finger protein 1 and a reduction in production of ROS (12–15). Moreover, muscle atrophy was reversed by reintroduction of vitamin C into the diet in 1 of these studies (12).

### Deficiency and low intakes

In general terms, the prevalence of vitamin C deficiency is greater in men than in women and is high in low-income and vulnerable populations in care (38–41). Whereas the prevalence of very low plasma concentrations of vitamin C, indicative of scurvy, in our cohort population was 0.9% in men and 0.2% in women, more than one-third of men (35%) and one-sixth of women (17%) were vitamin C insufficient. Within the United States 14% of men and 8% of women of a similar age to those in our study are deficient (42), and the prevalence of insufficiency in the UK population, as a whole, is 57% in men and 39% in women (41). In terms of the dietary guidelines for vitamin C, these range from the older UK guidelines with a recommended UK estimated average requirement (EAR) of 40 mg/d to the EFSA guidelines of 90 mg/d in men and 80 mg/d in women (16, 43, 44). Within our cohort 11% of men and 10% of women had intakes of vitamin C below the UK EAR, with the equivalent proportions for the EFSA guidelines being 59% in men and 47% in women. In our study, only those people in Q4 and Q5 of vitamin C intake consumed amounts at or above the EFSA guidelines, indicating that 60% of the population were consuming insufficient vitamin C.

Given that we found potentially clinically significant effects of insufficient dietary and circulating vitamin C on FFM, strategies to reduce the proportion of individuals with insufficient vitamin C status by increasing vitamin C intake may be beneficial for skeletal muscle health at a population level. This suggestion is also supported by a previous dietary supplementation intervention study where increased intake of vitamin C caused an increase in concentration of vitamin C in plasma, but also in the vastus lateralis (the largest of the quadriceps leg muscles) (45).

Analysis of dietary data for our cohort showed that for both men and women the greatest contributors to vitamin C intake were fruits, vegetables, potatoes, and fruit juices (Figure 4). Although such foods are typically readily available and easy to prepare and small increases in daily consumption should be achievable, limitations in income, access, and availability exist in at-risk populations. In our cohort the mean interquintile differences in vitamin C intakes for men and women were 133 and 132 mg/d, respectively, a >4-fold difference between Q1 and Q5. The individuals in Q1, consuming a mean of ∼40 mg/d, would need to consume the equivalent of 1 citrus fruit (e.g., an orange), a glass of apple juice, and a vegetable accompaniment with a meal (e.g., cabbage or broccoli) to achieve the same vitamin C intake as individuals in Q5.

### Strengths and limitations

We believe our study is a significant advance on previous research in this area and has a number of particular strengths. This is the largest study to examine concurrent associations of dietary and plasma vitamin C with measures of skeletal muscle (FFM% and FFM_BMI_) in both men and women of both middle and older age (20–25). Dietary intakes were calculated using 7-d food diaries. This method provides more accurate estimates of vitamin C than FFQs, which have been shown to systematically estimate intakes of vitamin C as ∼50% higher than the time-dependent methods of 7-d diaries or 24-h recalls (17, 46). The use of plasma vitamin C in this study is advantageous because this measurement accounts for factors that affect the absorption and metabolism of dietary vitamin C (e.g., current smoking habit). This helps to validate our findings because plasma vitamin C is a good biomarker of vegetable and fruit consumption and avoids the potential reporting bias in using dietary intake records (17, 18). However, blood samples were taken from nonfasted participants and thus steady-state vitamin concentrations may have been overestimated. We adjusted for recognized risk factors and lifestyle variables, including protein intake, that are known to affect skeletal muscle mass. To acknowledge any potential contribution of vitamin C supplementation, in parallel analyses we tested models with and without supplementation data and found that supplementation did not materially alter our findings. A general limitation of our study is the cross-sectional design, which means we cannot infer a causative link between vitamin C intake and skeletal muscle mass measures, and we cannot assess the temporality of associations. Dietary data were derived from food intake only, and thus excluded supplements; however, when we tested the quantitative contribution of vitamin C supplementation there were no significant changes to our findings. BIA was used to measure body composition, which is regarded to be less precise than DXA measurements. However in healthy individuals, BIA is considered as an accurate, practical, noninvasive data collection method, for measurements of body composition when compared with the more precise DXA method (47, 48). For sarcopenia diagnosis both low muscle mass and low muscle function are expected, but presarcopenia (itself a major risk factor for sarcopenia) is characterized by low muscle mass without overt effects on muscle function (3). The metabolic consequences of loss of skeletal muscle are also potentially important for sarcopenia (4). Thus, although muscle function data were not available for our analyses, our study investigating FFM as an estimate of skeletal muscle mass is still valuable for understanding risk of sarcopenia. Our analyses were conducted on a generally healthy population cohort and we did not adjust our analyses for chronic disease status which may influence sarcopenia outcomes. Lastly, the EPIC-Norfolk cohort is a population from a geographically defined area, with little outward migration, and consisting mainly of Caucasian participants, thus our results may be less applicable to different ethnic groups.

Overall, our findings suggest that consuming a diet high in vitamin C has potential for protection of skeletal muscle health during aging and thereby provides reinforcement to the benefits of following healthy eating guidelines by consuming sufficient fruits and vegetables. Further studies are required that include longitudinal analyses and intervention trials to investigate the long-term effects of increasing dietary or supplemental vitamin C on skeletal muscle health during aging.

### Conclusion

This study has shown significant positive associations between both dietary and circulating vitamin C and measures of skeletal muscle in a large cohort of free-living middle- and older-aged men and women. These results suggest that ensuring sufficient dietary vitamin C intake, by promoting a diet rich in fruits and vegetables, may help to reduce age-related loss of skeletal muscle and thus have wide-reaching public health benefit.

## Supplementary Material

nxaa221_Supplemental_Tables

## Notes

The European Prospective Investigation into Cancer and Nutrition–Norfolk study was supported by Medical Research Council grant G9502233 and Cancer Research UK grants SP2024-0201 and SP2024-0204.

Author disclosures: The authors report no conflicts of interest.

Supplemental Tables 1–3 are available from the “Supplementary data” link in the online posting of the article and from the same link in the online table of contents at https://academic.oup.com/jn/.

LNL and RPGH contributed equally to this work.

Abbreviations used: BIA, bioelectrical impedance analysis; EAR, estimated average requirement; EFSA, European Food Safety Authority; EPIC, European Prospective Investigation into Cancer and Nutrition; FFM, fat-free mass; FFM_BMI_, fat-free mass standardized by BMI; FFM%, fat-free mass percentage; HRT, hormone replacement therapy; Q, quintile; ROS, reactive oxygen species.
