# Supplementary material for: Lower Dietary and Circulating Vitamin C in Middle- and Older-Aged Men and Women Are Associated with Lower Estimated Skeletal Muscle Mass
Source: J Nutr. 2020 Aug 27;150(10):2789–98. doi: 10.1093/jn/nxaa221 (PMC7549302; doi:10.1093/jn/nxaa221)
Supplement: nxaa221_Supplemental_Tables [file nxaa221_supplemental_tables.docx]

**Supplementary Table 1.** Participant characteristics of the EPIC-Norfolk cohort population stratified by sex for the dietary vitamin C (n=14339) and the plasma vitamin C groups (n=13065) and also stratified by age group.^1^

|  | **Dietary vitamin C group** | | | | **Plasma vitamin C group** | | | |
| --- | --- | --- | --- | --- | --- | --- | --- | --- |
|  | **Men** | | **Women** | | **Men** | | **Women** | |
| **Characteristic** | **<65y (n=3477)** | **≥65y (n=2873)** | **<65y (n=4887)** | **≥65y (n=3102)** | **<65y (n=3209)** | **≥65y (n=2644)** | **<65y (n=4413)** | **≥65y (n=2799)** |
| Age, *y* | 56.0 (5.4) | 71.3 (4.2) | 55.5 (5.3) | 71.1 (4.2) | 56.0 (5.3) | 71.2 (4.2) | 55.5 (5.3) | 71.0 (4.2) |
| BMI, *kg/m^2^* | 26.6 (3.1) | 26.9 (3.0) | 25.8 (3.7) | 26.5 (3.7) | 26.6 (3.0) | 26.8 (3.0) | 25.8 (3.7) | 26.5 (3.7) |
| Weight, *kg* | 81.9 (10.9) | 79.7 (10.5) | 68.1 (10.5) | 67.1 (10.3) | 81.9 (10.9) | 79.8 (10.4) | 68.1 (10.5) | 67.0 (10.2) |
| Height, *cm* | 175.3 (6.5) | 172.3 (6.3) | 162.3 (5.9) | 159.0 (5.8) | 175.4 (6.5) | 172.3 (6.3) | 162.4 (5.9) | 159.0 (5.9) |
| FFM percentage, *%* | 76.7 (5.8) | 76.7 (5.7) | 61.1 (8.3) | 60.6 (8.1) | 76.8 (5.8) | 76.7 (5.7) | 61.3 (8.3) | 60.6 (8.1) |
| FFM_BMI_, *kg/m^2^* | 2.4 (0.3) | 2.3 (0.2) | 1.6 (0.3) | 1.5 (0.2) | 2.4 (0.3) | 2.3 (0.2) | 1.6 (0.3) | 1.5 (0.2) |
| Vitamin C intake, *mg/day* | 90.4 (51.7) | 89.0 (49.6) | 93.7 (51.0) | 93.6 (48.7) | 90.7 (51.5) | 89.0 (49.3) | 94.0 (51.2) | 93.9 (48.9) |
| Plasma Vitamin C, *μmol/L* |  |  |  |  | 57.2 (19.8) | 56.5 (23.0) | 69.6 (23.6) | 67.8 (25.8) |
| Protein, *g/day* | 86.3 (18.3) | 79.9 (15.9) | 67.1 (13.9) | 64.7 (13.2) | 86.4 (18.3) | 80.0 (16.0) | 67.2 (14.0) | 64.8 (13.2) |
| Protein *% energy* | 14.7 (2.4) | 15.0 (2.4) | 15.4 (2.7) | 15.8 (2.8) | 14.7 (2.3) | 15.0 (2.4) | 15.4 (2.7) | 15.8 (2.8) |
| Energy intake, *kcal/day* | 2382 (509) | 2873 (464) | 1776 (386) | 1670 (355) | 2383 (509) | 2173 (466) | 1777 (388) | 1672 (354) |
| Smoking, *n* [%] |  |  |  |  |  |  |  |  |
| Current | 333 [9.6] | 209 [7] | 496 [10] | 200 [6] | 301 [9] | 194 [7] | 451 [10] | 178 [6] |
| Former | 1716 [49] | 1808 [63] | 1505 [31] | 1046 [34] | 1584 [49] | 1670 [63] | 1363 [31] | 949 [34] |
| Never | 1428 [41] | 856 [30] | 2886 [59] | 1856 [60] | 1324 [41] | 780 [30] | 2599 [59] | 1672 [60] |
| Physical activity, *n* [%] |  |  |  |  |  |  |  |  |
| Inactive | 719 [21] | 1017 [35] | 941 [19] | 1129 [36] | 646 [20] | 920 [35] | 827 [19] | 1002 [36] |
| Moderately inactive | 843 [24] | 752 [26] | 1563 [32] | 1037 [33] | 767 [24] | 691 [26] | 1430 [32] | 944 [34] |
| Moderately Active | 979 [28] | 611 [21] | 1333 [27] | 600 [19] | 912 [28] | 573 [22] | 1190 [27] | 547 [20] |
| Active | 936 [27] | 493 [17] | 1050 [21] | 336 [11] | 884 [28] | 460 [17] | 966 [22] | 306 [11] |
| Corticosteroid use, *n* [%] | 106 [3] | 158 [5] | 204 [4] | 203 [7] | 96 [3] | 141 [5] | 185 [4] | 180 [6] |
| Menopausal status, *n* [%] |  |  |  |  |  |  |  |  |
| Premenopausal |  |  | 473 [10] | 2 [0.06] |  |  | 425 [10] | 2 [0.07] |
| Perimenopausal <1y |  |  | 266 [5] |  |  |  | 238 [5] |  |
| Perimenopausal 1-5y |  |  | 1381 [28] | 18 [0.6] |  |  | 1239 [28] | 17 [6] |
| Postmenopausal >5y |  |  | 2767 [57] | 3082 [99] |  |  | 2511 [57] | 2780 [99] |
| HRT, *n* [%] |  |  |  |  |  |  |  |  |
| Current |  |  | 1456 [30] | 248 [8] |  |  | 1323 [30] | 220 [8] |
| Former |  |  | 1036 [21] | 395 [13] |  |  | 926 [21] | 362 [13] |
| Never |  |  | 2395 [49] | 2459 [79] |  |  | 2164 [49] | 2217 [79] |
| Statins use, *n* [%] | 153 [4] | 194 [7] | 103 [2] | 187 [6] | 141 [4] | 177 [22] | 90 [2] | 167 [6] |
| Days diary completed | 6.6 (1.4) | 6.9 (0.9) | 6.8 (1.1) | 6.9 (0.8) | 6.6 (1.3) | 6.9 (0.8) | 6.8 (1.1) | 6.9 (0.8) |
| Vitamin C supplements, *n* [%] | 354 [38] | 341 [32] | 934 [43] | 491 [35] | 315 [36] | 318 [32] | 850 [43] |  |
| Social class, *n* [%] |  |  |  |  |  |  |  |  |
| Professional | 283 [8] | 240 [8] | 348 [7] | 198 [6] | 261 [8] | 218 [8] | 310 [7] | 176 [6] |
| Managerial | 1433 [41] | 1154 [40] | 1859 [38] | 1091 [35] | 1327 [41] | 1068 [40] | 1689 [38] | 979 [35] |
| Skilled non-manual | 407 [12] | 390 [14] | 843 [17] | 711 [23] | 363 [11] | 360 [14] | 761 [17] | 652 [23] |
| Skilled manual | 818 [24] | 604 [21] | 1043 [21] | 534 [17] | 758 [24] | 554 [21] | 941 [21] | 485 [17] |
| Semi-skilled | 419 [12] | 362 [13] | 591 [12] | 359 [12] | 393 [12] | 334 [13] | 538 [12] | 317 [11] |
| Non-skilled | 78 [2] | 71 [2] | 143 [3] | 124 [4] | 70 [2] | 63 [2] | 55 [1] | 115 [4] |

^1^ Differences between men and women. Values are mean (SD) or n [%]. Vitamin C group characteristics at first health check. Plasma vitamin C characteristic at second health check (time of BIA measures). Fat Free Mass Percentage (FFM%), Fat Free Mass standardised by BMI (FFM_BMI_)

**Supplementary Table 2.** Associations between quintiles of dietary vitamin C and fat free mass, adjusted mean values, in men and women aged 42-82 years^1^

| **Men (n=6350)** | **FFM%** | | | | **FFM_BMI_** | | | |
| --- | --- | --- | --- | --- | --- | --- | --- | --- |
|  | **<65y (n=3477)** | | **≥65y (n=2873)** | | **<65y (n=3477)** | | **≥65y (n=2873)** | |
| Vitamin C Quintile | Unadjusted | Adjusted | Unadjusted | Adjusted | Unadjusted | Adjusted | Unadjusted | Adjusted |
| 1 | 75.9±0.23 | 76.2±0.17 | 76.6±0.25 | 76.4±0.19 | 2.30±0.01 | 2.32±0.01 | 2.24±0.01 | 2.27±0.01 |
| 2 | 76.8±0.22 | 76.7±0.16 | 77.0±0.24 | 76.6±0.19 | 2.36±0.01 | 2.36±0.01 | 2.27±0.01 | 2.27±0.01 |
| 3 | 76.9±0.22 | 76.8±0.16 | 76.5±0.23 | 76.6±0.19 | 2.38±0.01 | 2.37±0.01 | 2.28±0.01 | 2.27±0.01 |
| 4 | 76.6±0.23 | 76.6±0.16 | 77.1±0.24 | 77.1±0.19 | 2.37±0.01 | 2.36±0.01 | 2.31±0.01 | 2.30±0.01 |
| 5 | 77.3±0.21 | 77.2±0.16 | 76.5±0.23 | 76.9±0.19 | 2.41±0.01 | 2.39±0.01 | 2.30±0.01 | 2.29±0.01 |
| Q5-Q1 difference ^2^ | 1.34 (0.72, 1.95) | 1.04 (0.57, 1.51) | -0.08 (-0.74, 0.58) | 0.43 (-0.11, 0.97) | 0.11 (0.08, 0.14) | 0.07 (0.04, 0.10) | 0.06 (0.03, 0.09) | 0.03 (-0.001, 0.06) |
| % difference ^3^ | 1.76 | 1.36 | -0.10 | 0.57 | 4.80 | 2.98 | 2.63 | 1.20 |
| P trend | 0.001 | <0.001 | 0.663 | 0.05 | <0.001 | <0.001 | <0.001 | 0.01 |
| **Women (n=7989)** | **FFM%** | | | | **FFM_BMI_** | | | |
|  | **<65y (n=4887)** | | **≥65y (n=3102)** | | **<65y (n=4887)** | | **≥65y (n=3102)** | |
| Vitamin C Quintile | Unadjusted | Adjusted | Unadjusted | Adjusted | Unadjusted | Adjusted | Unadjusted | Adjusted |
| 1 | 60.2±0.28 | 60.6±0.21 | 59.9±0.34 | 59.9±0.27 | 1.58±0.01 | 1.59±0.01 | 1.50±0.01 | 1.51±0.01 |
| 2 | 61.3±0.27 | 61.0±0.20 | 60.5±0.32 | 60.6±0.27 | 1.61±0.01 | 1.61±0.01 | 1.54±0.01 | 1.54±0.01 |
| 3 | 61.2±0.26 | 61.3±0.20 | 60.7±0.33 | 60.4±0.26 | 1.63±0.01 | 1.63±0.01 | 1.53±0.01 | 1.52±0.01 |
| 4 | 61.2±0.25 | 61.1±0.20 | 60.6±0.32 | 60.7±0.27 | 1.62±0.01 | 1.61±0.01 | 1.54±0.01 | 1.54±0.01 |
| 5 | 61.8±0.26 | 61.7±0.20 | 61.1±0.31 | 61.3±0.27 | 1.64±0.01 | 1.64±0.01 | 1.56±0.01 | 1.56±0.01 |
| Q5-Q1 difference | 1.52 (0.78, 2.26) | 1.13 (0.55, 1.71) | 1.22 (0.32, 2.12) | 1.31 (0.55, 2.06) | 0.07 (0.04, 0.09) | 0.04 (0.02, 0.07) | 0.06 (0.04, 0.09) | 0.05 (0.02, 0.08) |
| % difference | 2.52 | 1.86 | 2.04 | 2.18 | 4.17 | 2.79 | 4.14 | 3.32 |
| P trend | 0.001 | <0.001 | 0.015 | 0.001 | <0.001 | <0.001 | <0.001 | 0.001 |

^1^ Values are presented as means ± SEM. The P-trend was calculated using ANCOVA.

^2^ Q5-Q1 calculates the absolute difference between the means of quintile(Q) 5 and Q1, with 95% confidence intervals.

^3^ % difference calculates the percentage difference between the means of Q5 and Q1.

Adjusted model includes age, total energy, protein intake as a percentage of total energy, Estimated Energy Requirement, smoking status, physical activity, corticosteroid use, menopausal status, HRT use, statins use, number of days participant filled out in the diary, social class.

**Dietary Vitamin C intake (mean ± SD; mg/day, median) by vitamin C** **quintiles.** *Men<65*years: Q1 (n=685) 36.4±9.6, 38.5; Q2(n=693) 57.8±5.3, 57.7; Q3 (n=699) 78.4±6.6, 78.3; Q4 (n=699) 106.8±10.2, 105.8; Q5 (n=701) 171.0±46.7, 156.7. *Women<65years:* Q1(n=975) 38.7±9.5, 40.6; Q2(n=995) 62.7±5.9, 62.6; Q3(n=961) 83.9±6.8,83.6; Q4(n=979) 111.5±9.9, 111.2; Q5(n=977) 172.0±47.2, 159.2. *Men≥65 years:* Q1(n=585) 36.8±9.0, 38.5; Q2(n=577) 57.9±5.5, 57.7; Q3(n=571) 78.4±6.7, 78.3; Q4 (n=571) 106.1±10.0, 105.8; Q5(n=569) 167.7±42.2, 156.7*. Women≥65 years*: Q1(n=623) 39.2±9.3, 40.6; Q2(n=603) 62.6±5.9, 62.6; Q3 (n=637) 83.8±6.7, 83.6; Q4 (n=619) 112.0±9.8, 111.2; Q5(n=620) 170.3±36.8, 159.2.

**Supplementary Table 3**. Associations between plasma vitamin C categories and fat free mass, adjusted mean values, in men and women aged between 42-82years stratified by age group^1^

| **Men (n=5853)** | **FFM%** | | | | **FFM_BMI_** | | | |
| --- | --- | --- | --- | --- | --- | --- | --- | --- |
|  | **<65y (n=3209)** | | **≥65y (n=2644)** | | **<65y (n=3209)** | | **≥65y (n=2644)** | |
| Vitamin C category | Unadjusted | Adjusted | Unadjusted | Adjusted | Unadjusted | Adjusted | Unadjusted | Adjusted |
| Insufficient <50*μmol/L* | 75.5±0.18 | 75.6±0.18 | 76.3±0.19 | 76.3±0.18 | 2.32±0.01 | 2.33±0.01 | 2.26±0.01 | 2.26±0.01 |
| Sufficient ≥50*μmol/L* | 77.4±0.12 | 77.3±0.12 | 77.0±0.14 | 77.0±0.14 | 2.39±0.01 | 2.38±0.01 | 2.30±0.01 | 2.29±0.01 |
| Absolute difference ^2^ | 1.87 (1.44, 2.29) | 1.77 (1.34, 2.19) | 0.64 (0.19, 1.08) | 0.71 (0.27, 1.16) | 0.07 (0.05, 0.09) | 0.06 (0.04, 0.08) | 0.04 (0.02, 0.06) | 0.03 (0.02, 0.05) |
| % difference ^3^ | 2.47 | 2.34 | 0.83 | 0.94 | 3.02 | 2.49 | 1.69 | 1.52 |
| P value | <0.001 | <0.001 | 0.005 | 0.002 | <0.001 | <0.001 | <0.001 | <0.001 |
| **Women (n=7212)** | **FFM%** | | | | **FFM_BMI_** | | | |
|  | **<65y (n=4413)** | | **≥65y (n=2799)** | | **<65y (n=4413)** | | **≥65y (n=2799)** | |
| Vitamin C category | Unadjusted | Adjusted | Unadjusted | Adjusted | Unadjusted | Adjusted | Unadjusted | Adjusted |
| Insufficient <50*μmol/L* | 59.1±0.35 | 59.0±0.32 | 59.6±0.26 | 59.4+0.35 | 1.56±0.01 | 1.56±0.01 | 1.50±0.01 | 1.50±0.01 |
| Sufficient ≥50*μmol/L* | 61.6±0.13 | 61.6±0.13 | 60.9±0.10 | 60.9±0.17 | 1.63±0.004 | 1.63±0.004 | 1.54±0.003 | 1.54±0.01 |
| Absolute difference | 2.55 (1.87, 3.22) | 2.61 (1.93, 3.28) | 1.27 (0.52, 2.03) | 1.49 (0.73, 2.25) | 0.07 (0.05, 0.10) | 0.07 (0.05, 0.09) | 0.05 (0.02, 0.07) | 0.05 (0.02, 0.07) |
| % difference | 4.31 | 4.40 | 2.14 | 2.51 | 4.75 | 4.68 | 3.07 | 3.11 |
| P value | <0.001 | <0.001 | 0.001 | <0.001 | <0.001 | <0.001 | <0.001 | <0.001 |

^1^ Values are presented as means ± SEM. P values were calculated using ANCOVA comparing the two vitamin C categories.

^2^ Absolute difference calculates the difference between means of the two categories, with 95% confidence intervals.

^3^ % difference calculates the percentage difference between means of the two categories.

Adjusted model adjusted for age, smoking status, physical activity, corticosteroid use, menopausal status, HRT use, statins use, and social class.

**Dietary Vitamin C intake (mean ± SD; mg/day, median) by vitamin C categories.** *Men<65 years*: Insufficient (n=1038) 72.7±40.7, 62.8; Sufficient (n=2171), 99.2±53.9, 87.8. *Men≥65 years:* Insufficient (n=997) 73.1±38.6, 64.0; Sufficient (n=1647) 98.7±54.4, 88.0. *Women<65 years*: Insufficient (n=668) 73.5±40.5, 64.0; Sufficient (n=3745), 97.7±52.0, 87.5. *Women≥65 years:* Insufficient (n=544) 72.6±40.8, 63.7; Sufficient (n=2255) 99.1±49.3, 89.9. **Plasma Vitamin C (mean ± SD; μmol/L, median) by vitamin C categories** *Men<65 years*: Insufficient (n=1038) 36.5±10.5, 39; Sufficient (n=2171), 67.1±15.0, 64. *Men≥65 years:* Insufficient (n=997) 35.2±11.1, 38; Sufficient (n=1647) 69.4±18.2, 65. *Women<65 years*: Insufficient (n=668) 38.5±9.6, 41; Sufficient (n=3745), 75.2±20.9, 72. *Women≥65 years:* Insufficient (n=544) 35.5±10.4, 38; Sufficient (n=2255) 75.6.1±22.1, 72.
